# The Impact of Preoperative Anemia on Complications After Total Shoulder Arthroplasty

**DOI:** 10.5435/JAAOSGlobal-D-20-00136

**Published:** 2021-01-19

**Authors:** Kevin I. Kashanchi, Alireza K. Nazemi, David E. Komatsu, Edward D. Wang

**Affiliations:** From the Renaissance School of Medicine at Stony Brook University (Mr. Kashanchi), and the Department of Orthopaedics, Stony Brook University (Dr. Nazemi, Dr. Komatsu, and Dr. Wang), Stony Brook, NY.

## Abstract

This study investigated the relationship between varying levels of preoperative anemia and postoperative complications within 30 days of total shoulder arthroplasty (TSA).

**Methods:**

All patients who underwent TSA from 2015 to 2017 were queried from the American College of Surgeons National Surgical Quality Improvement database. Patients were categorized based on preoperative hematocrit levels: normal (>39% for men and >36% for women), mild anemia (29% to 39% for men and 29% to 36% for women), and severe anemia (<29% for both men and women).

**Results:**

A total of 10,547 patients were included in the study. Of these patients, 1,923 patients were (18.2%) in the mild anemia cohort and 146 (1.4%) were in the severe anemia cohort. Mild anemia was identified as a significant predictor of any complication (odds ratio [OR] 2.74, *P* < 0.001), stroke/cerebrovascular accident (OR 6.79, *P* = 0.007), postoperative anemia requiring transfusion (OR 6.58, *P* < 0.001), nonhome discharge (OR 1.79, *P* < 0.001), readmission (OR 1.63, *P* < 0.001), and return to the surgical room (OR 1.60, *P* = 0.017). Severe anemia was identified as a significant predictor of any complication (OR 4.31, *P* < 0.001), renal complication (OR 13.78, *P* < 0.001), postoperative anemia requiring transfusion (OR 5.62, *P* < 0.001), and nonhome discharge (OR 2.34, *P* < 0.001).

**Conclusion:**

Preoperative anemia status is a risk factor for complications within 30 days of TSA.

In the past decade, the indications for total shoulder arthroplasty (TSA) have evolved to encompass glenohumeral arthritis, proximal humerus fractures, and severe rotator cuff pathology.^1,2,3,4,5^ Approximately 100,000 TSAs are done each year in the United States, with an estimated 10.6% annual growth rate.^2,6^ Although TSA has been associated with good functional outcomes and high patient satisfaction ratings, the literature suggests a short-term complication rate between 2.5% and 8%.^7,8,9,10,11^

Anemia is a common condition estimated to affect approximately 11% amount of the population older than the age of 65 years.^12,13^ Within the context of TSA, preoperative anemia has been identified as a predictor for many postoperative complications including postoperative anemia requiring transfusion, extended length of stay, and pulmonary embolism.^7,14,15,16,17,18,19,20^ In all of these studies, preoperative anemia was treated as a binary variable, and the relationship between the severity of preoperative anemia status and its relationship to postoperative complications was not elucidated. The present study used a large national database to investigate the relationship between varying levels of preoperative anemia and postoperative complications within 30 days of TSA.

The purpose of this study was to identify whether preoperative anemia, after controlling for associated patient demographics and comorbidities, is a risk factor for short-term complications after TSA.

## Methods

All patients who underwent TSA between 2015 and 2017 were queried from the American College of Surgeons National Surgical Quality Improvement Program (ACS NSQIP) database. The retrospective data included in this study were deidentified, and the study was exempt from approval by our University's Institutional Review Board. Between 2015 and 2017, the ACS NSQIP database collected patient data and 274 different variables from over 600 community and academic hospitals. These data were collected by trained, certified surgical clinical reviewers, and the database is regularly audited by the ACS to ensure high quality data.^21^

Patients who underwent TSA were identified by Current Procedural Terminology code 23472, which included both anatomic and reverse TSAs. Patients younger than the age of 18 years were excluded from the analysis. Patient demographics including age, sex, body mass index (BMI), American Society of Anesthesiologists (ASA) physical status classification class, smoking status, functional status, steroid use, and preoperative medical comorbidities were collected. Dependent functional status included patients who were either partially or totally dependent. An ASA status of three or greater indicated a patient with severe systemic disease. Smoking status was defined as current smoker within one year.

Patients were grouped into four cohorts based on the preoperative hematocrit levels. Normal preoperative hematocrit levels (>39% for men and >36% for women), mild anemia (29% to 39% for men and 29% to 36% for women), and severe anemia (<29% for both men and women). A combined anemia cohort included both mild and severe anemia cohorts. Preoperative anemia was defined in accordance with the World Health Organization's sex-based criteria, which defines anemia as a hematocrit <39% in men and a hematocrit <36% in women.^22^ Anemic patients were further stratified into mild and severe anemia cohorts according to their preoperative hematocrit levels, as defined by a previous literature report.^23^ The complications included in the analysis were cardiac complications, renal complications, respiratory complications, deep vein thrombosis, stroke or cerebrovascular accident (CVA), sepsis, wound infection, wound dehiscence, urinary tract infection (UTI), postoperative anemia requiring transfusion, nonhome discharge destination, hospital readmission, and return to the surgical room within 30 days postoperatively.

SPSS software version 26.0 (IBM) was used to do the statistical analyses. Bivariate analysis compared patient demographics, comorbidities, and procedural characteristics between the cohorts. Multivariate logistic regression was adjusted for notably associated variables to investigate the impact of preoperative anemia status on postoperative complications. Rates of complications were also compared between cohorts using bivariate analysis. Statistical significance was set at *P* < 0.05.

## Results

From 2015 to 2017, a total of 10,547 patients who underwent TSA were included in the study. Of these patients, 2,069 patients (19.6%) were in the combined anemia cohort, 1,923 patients (18.2%) were in the mild anemia cohort, and 146 patients (1.4%) were in the severe anemia cohort. In comparison to the normal hematocrit cohort, the anemia cohort had a greater proportion of patients who were women, older than the age of 70 years, and were afflicted by comorbidities including diabetes, chronic obstructive pulmonary disease, and hypertension. In addition, a greater proportion of patients with anemia were functionally dependent, used corticosteroids, had an ASA ≥3, and had a BMI <25 (Table 1).

**Table 1:** Comparison of Patient Demographic, Comorbidity, and Procedural Characteristics Between Anemic Cohorts

Characteristic	All Patients (n = 10,547)	Preoperative Hematocrit				*P* Value	*P* Value	*P* Value
		Normal (n = 8,478)	Any Anemia (n = 2,069)	Mild Anemia (n = 1,923)	Severe Anemia (n = 146)	Anemia Versus No Anemia	Mild Anemia Versus No Anemia	Mild Anemia Versus Severe
Age								
<60	14.9% (1,574)	16.1% (1,363)	10.2% (211)	10.0% (192)	13.0% (19)	**<0.001**	**<0.001**	0.255
60-69	34.2% (3,610)	35.7% (3,026)	28.2% (584)	28.2% (543)	28.1% (41)	**<0.001**	**<0.001**	1.000
70-79	37.5% (3,956)	36.6% (3,104)	41.2% (852)	41.2% (792)	41.1% (60)	**<0.001**	**<0.001**	1.000
>80	13.3% (1,407)	11.6% (985)	20.4% (422)	20.6% (396)	17.8% (26)	**<0.001**	**<0.001**	0.457
Sex						**0.003**	**0.029**	**0.003**
Male	44.0% (4,636)	44.7% (3,787)	41.0% (849)	41.9% (806)	29.5% (43)			
Female	56.0% (5,911)	55.3% (4,691)	59.0% (1,220)	58.1% (1,117)	70.5% (103)			
BMI								
<25	16.5% (1,741)	15.3% (1,300)	21.3% (441)	21.0% (403)	26.0% (38)	**<0.001**	**<0.001**	0.172
25-30	32.4% (3,412)	32.3% (2,739)	32.5% (673)	32.2% (620)	36.3% (53)	0.855	0.978	0.315
30-35	26.5% (2,793)	27.0% (2,292)	24.2% (501)	24.2% (465)	24.7% (36)	**0.009**	**0.011**	0.920
35-40	13.8% (1,456)	14.2% (1,205)	12.1% (251)	12.5% (241)	6.8% (10)	**0.015**	0.059	**0.047**
>40	10.9% (1,145)	11.1% (942)	9.8% (203)	10.1% (194)	6.2% (9)	0.091	0.210	0.148
ASA						**<0.001**	**<0.001**	0.774
1 or 2	42.5% (4,482)	46.1% (3,908)	27.7% (574)	27.7% (532)	28.8% (42)			
≥3	57.5% (6,065)	53.9% (4,570)	72.3% (1,495)	72.3% (1,391)	71.2% (104)			
Diabetes						**<0.001**	**<0.001**	1.000
Yes	18.3% (1,927)	16.4% (1,388)	26.1% (539)	26.1% (501)	26.0% (38)			
No	81.7% (8,620)	83.6% (7,090)	73.9% (1,530)	73.9% (1,422)	74.0% (108)			
COPD						**<0.001**	**<0.001**	0.554
Yes	6.9% (729)	6.3% (538)	9.2% (191)	9.4% (180)	7.5% (11)			
No	93.1% (9,818)	93.7% (7,940)	90.8% (1,878)	90.6% (1,743)	92.5% (135)			
Functional dependence						**<0.001**	**<0.001**	1.000
Yes	2.3% (241)	1.8% (155)	4.2% (86)	4.2% (80)	4.1% (6)			
No	97.7% (10,306)	98.2% (8,323)	95.8% (1,983)	95.8% (1,843)	95.9% (140)			
Current smoker						**0.008**	**0.003**	0.145
Yes	11.2% (1,185)	11.6% (987)	9.6% (198)	9.3% (179)	13.0% (19)			
No	88.8% (9,362)	88.4% (7,491)	90.4% (1,871)	90.7% (1,744)	87% (127)			
Steroid use						**<0.001**	**<0.001**	0.410
Yes	5.2% (548)	4.7% (397)	7.3% (151)	7.2% (138)	8.9% (13)			
No	94.8% (9,999)	95.3% (8,081)	92.7% (1,918)	92.8% (1,785)	91.1% (133)			
Hypertension						**<0.001**	**<0.001**	0.051
Yes	67.7% (7,141)	65.3% (5,540)	77.4% (1,601)	77.9% (1,498)	70.5% (103)			
No	32.3% (3,406)	34.7% (2,938)	22.6% (468)	22.1% (425)	29.5% (43)			
Anesthesia						**0.001**	**0.001**	0.215
General	96.9% (10,215)	96.6% (8,188)	98.0% (2,027)	98.1% (1,886)	96.6% (141)			
Regional	3.1% (332)	3.4% (290)	2.0% (42)	1.9% (37)	3.4% (5)			

ASA = American Society of Anesthesiologists; BMI = body mass index; COPD = chronic obstructive pulmonary disease; HTN = hypertension.

Bold values in Tables 1-3 indicate statistically significant values.

Increasing rates of any complication progressing from the normal hematocrit cohort (3.1%), to the mild anemia cohort (9.5%), to the severe anemia cohort (30.8%; Table 2) existed. In addition, increasing rates of postoperative anemia requiring transfusion (0.84%, 6.4%, 28.1%) and nonhome discharge (8.4%, 18.3%, 32.9%) from normal hematocrit to mild to severe anemia levels, respectively, existed. In comparison to the normal hematocrit level cohort, patients in the mild anemia cohort had a higher rate of any complication (3.1% versus 9.5%, *P* < 0.001), cardiac complication (0.26% versus 0.62%, *P* = 0.023), stroke/CVA (0.04% versus 0.36%, *P* = 0.001), sepsis (0.18% versus 0.47%, *P* = 0.030), postoperative anemia requiring transfusion (0.84% versus 6.4%, *P* < 0.001), nonhome discharge (8.4% versus 18.3%, *P* < 0.001), hospital readmission (2.6% versus 5.1%, *P* < 0.001), and return to the surgical room (1.2% versus 2.1%, *P* < 0.001). In comparison to the normal hematocrit level cohort, patients in the combined anemic cohort had a higher rate of any complication (3.1% versus 11.0%, *P* < 0.001), cardiac complication (0.26% versus 0.68%, *P* = 0.10), renal complication (0.12% versus 0.43%, *P* = 0.006), stroke/CVA (0.04% versus 0.34%, *P* = 0.001), sepsis (0.18% versus 0.43%, *P* = 0.037), UTI (0.58% versus 1.1%, *P* = 0.023), postoperative anemia requiring transfusion (0.84% versus 8.0%, *P* < 0.001), nonhome discharge (8.4% versus 19.3%, *P* < 0.001), hospital readmission (2.6% versus 5.2%, *P* < 0.001), and return to the surgical room (1.2% versus 2.1%, *P* = 0.001; Table 2). In comparison to the mild anemia cohort, patients with severe anemia had a higher rate of any complication (9.5% versus 30.8%, *P* < 0.001), renal complication (0.26% versus 2.7%, *P* = 0.002), postoperative anemia requiring transfusion (6.4% versus 28.1%, *P* < 0.001), and nonhome discharge (18.3% versus 32.9%, *P* < 0.001; Table 2).

**Table 2:** Comparison of Complication Rates Between Anemia Cohorts

Factor	All Patients (n = 10,547)	Preoperative Hematocrit				*P* Value	*P* Value	*P* Value
		Normal (n = 8,478)	Any Anemia (n = 2,069)	Mild Anemia (n = 1,923)	Severe Anemia (n = 146)	Anemia Versus No Anemia	Mild Anemia Versus No Anemia	Mild Anemia Versus Severe
Any complication	4.6% (489)	3.1% (261)	11.0% (228)	9.5% (183)	30.8% (45)	**<0.001**	**<0.001**	**<0.001**
Cardiac complication	0.34% (36)	0.26% (22)	0.68% (14)	0.62% (12)	1.4% (2)	**0.010**	**0.023**	0.259
Renal complication	0.18% (19)	0.12% (10)	0.43% (9)	0.26% (5)	2.7% (4)	**0.006**	0.174	**0.002**
Respiratory complication	0.96% (101)	0.87% (74)	1.3% (27)	1.2% (24)	2.1% (3)	0.074	0.146	0.433
DVT	0.35% (37)	0.33% (28)	0.43% (9)	0.47% (9)	0% (0)	0.532	0.394	1.000
Stroke/CVA	0.09% (10)	0.04 (3)	0.34% (7)	0.36% (7)	0% (0)	**0.001**	**0.001**	1.000
Sepsis	0.23% (24)	0.18% (15)	0.43% (9)	0.47% (9)	0% (0)	**0.037**	**0.030**	1.000
Wound infection	0.44% (46)	0.39% (33)	0.63% (13)	0.62% (12)	0.68% (1)	0.139	0.176	0.615
Wound dehiscence	0.04% (4)	0.05% (4)	0% (0)	0% (0)	0% (0)	1.000	1.000	—
UTI	0.67% (71)	0.58% (49)	1.1% (22)	0.94% (18)	2.7% (4)	**0.023**	0.083	0.064
Postoperative transfusion	2.25% (237)	0.84% (71)	8.0% (165)	6.4% (124)	28.1% (41)	**<0.001**	**<0.001**	**<0.001**
Nonhome discharge	10.5% (1,110)	8.4% (710)	19.3% (400)	18.3% (352)	32.9% (48)	**<0.001**	**<0.001**	**<0.001**
Hospital readmission	3.1% (329)	2.6% (222)	5.2% (107)	5.1% (99)	5.5% (8)	**<0.001**	**<0.001**	0.846
Return to the surgical room	1.3% (142)	1.2% (98)	2.1% (44)	2.1% (40)	2.7% (4)	**0.001**	**0.002**	0.548

CVA = cerebrovascular accident; DVT = deep vein thrombosis; UTI = urinary tract infection.

Mild anemia was identified as a significant predictor of any complication (odds ratio [OR] 2.74, *P* < 0.001), stroke/CVA (OR 6.79, *P* = 0.007), postoperative anemia requiring transfusion (OR 6.58, *P* < 0.001), nonhome discharge (OR 1.79, *P* < 0.001), hospital readmission (OR 1.63, *P* < 0.001), and return to the surgical room (OR 1.60, *P* = 0.017; Table 3). Combined anemia was identified as a significant predictor of any complication (OR 3.26, *P* < 0.001), cardiac complication (OR 2.08, *P* = 0.038), renal complication (OR 2.74, *P* = 0.036), stroke/CVA (OR 6.44, *P* = 0.009), postoperative anemia requiring transfusion (OR 8.35, *P* < 0.001), nonhome discharge (OR 1.93, *P* < 0.001), hospital readmission (OR 1.63, *P* < 0.001), and return to the surgical room (OR 1.64, *P* = 0.009; Table 3). Severe anemia was identified as a significant predictor of any complication (OR 4.31, *P* < 0.001), renal complication (OR 13.78, *P* < 0.001), postoperative anemia requiring transfusion (OR 5.62, *P* < 0.001), and nonhome discharge (OR 2.34, *P* < 0.001; Table 3).

**Table 3:** Association Between the Severity of Preoperative Anemia and the Rate of Postoperative Complications

Factor	Anemia Versus No Anemia			Mild Anemia Versus No Anemia			Mild Anemia Versus Severe Anemia		
	OR	95% CI	*P* Value	OR	95% CI	*P* Value	OR	95% CI	*P* Value
Any complication	3.26	2.69-3.95	**<0.001**	2.74	2.24-3.37	**<0.001**	4.31	2.90-6.41	**<0.001**
Cardiac complications	2.08	1.04-4.14	**0.038**	1.91	0.93-3.94	0.08	2.67	0.57-12.43	0.212
Renal complications	2.74	1.07-7.03	**0.036**	1.61	0.52-4.92	0.408	13.78	3.27-58.02	**<0.001**
Respiratory complications	1.13	0.72-1.79	0.593	1.08	0.67-1.73	0.763	1.76	0.51-6.01	0.370
DVT	1.22	0.57-2.65	0.610	1.32	0.61-2.86	0.481	—	—	—
Stroke/CVA	6.44	1.59-26.11	**0.009**	6.79	1.68-27.5	**0.007**	—	—	—
Sepsis	1.83	0.78-4.31	0.166	1.94	0.82-4.55	0.130	—	—	—
Wound infection	1.90	0.97-3.72	0.061	1.83	0.92-3.67	0.085	1.11	0.13-9.25	0.921
Wound dehiscence	—	—	—	—	—	—	—	—	—
UTI	1.50	0.89-2.52	0.127	1.29	0.74-2.25	0.369	3.06	0.99	9.38
Postoperative transfusion	8.35	6.24-11.16	**<0.001**	6.58	5.85-8.93	**<0.001**	5.62	3.68-8.58	**<0.001**
Nonhome discharge	1.93	1.67-2.23	**<0.001**	1.79	1.54-2.08	**<0.001**	2.34	1.58-3.47	**<0.001**
Hospital readmission	1.63	1.27-2.08	**<0.001**	1.63	1.27-2.09	**<0.001**	1.08	0.51-2.29	0.842
Return to the surgical room	1.64	1.13-2.39	**0.009**	1.60	1.09-2.34	**0.017**	1.49	0.52-4.28	0.464

CI = confidence interval; CVA = cerebrovascular accident; DVT = deep vein thrombosis; OR = odds ratio; UTI = urinary tract infection.

## Discussion

This study identified preoperative anemia status as a notable risk factor for postoperative complications within 30 days of TSA. Even mildly decreased preoperative hematocrit levels (between 29% and 36% for women and between 29% and 39% for men) notably increased the risk of stroke/CVA, postoperative anemia requiring transfusion, nonhome discharge, hospital readmission, and return to the surgical room. In addition, severe anemia (hematocrit <29% in both men and women) was identified as a predictor of postoperative anemia requiring transfusion, nonhome discharge, and renal complications after TSA.

Previous investigations have identified that preoperative anemia is associated with an increased rate of complications after total joint arthroplasty.^24,25,26,27,28,29^ Viola et al^28^ identified an association between preoperative anemia, defined by hemoglobin <12 g/dL for women and <13 g/dL for men, and increased rates of postoperative complications and mortality after total knee arthroplasty and total hip arthroplasty. Similarly, Grosso et al^25^ identified preoperative anemia, defined by hematocrit <36%, as a notable risk factor for mortality, renal complications, respiratory complications, sepsis, wound infection, and UTI after THA. Within the realm of shoulder arthroplasty, preoperative anemia status has been previously identified as a risk factor for postoperative anemia requiring transfusion, extended length of stay, and pulmonary embolism.^7,14,15,16,17,18,19,20^ In particular, numerous studies have identified preoperative anemia as an independent risk factor for postoperative anemia requiring transfusion after TSA. In a retrospective study of 196 shoulder arthroplasty cases, Gruson et al^14^ found that the presence of preoperative anemia, defined by a hemoglobin <13 g/dL for men and a hemoglobin <12 g/dL for women, was associated with a 19-fold increase in the likelihood of postoperative anemia requiring transfusion. Similarly, in a study of 366 patients undergoing TSA, Hardy et al^15^ found that lower preoperative hemoglobin levels were associated with an increased likelihood for postoperative blood transfusions. Many studies using large national databases have also identified similar associations between anemia and postoperative blood transfusions. In a study using the Nationwide Inpatient Sample, Kandil et al^16^ identified preoperative anemia (OR 3.5, *P* < 0.01) as an independent risk factor for blood transfusions after TSA. However, to our knowledge, no study has investigated the relationship between varying degrees of preoperative anemia and its impact on postoperative complications after TSA.

Our study identified a notably increased complication rate (11.0%) among anemic patients versus normal hematocrit patients (3.1%) and a notable association between an increasing rate of complications and increasing severity of preoperative anemia. In addition, the overall complication rate in our study was 4.6%, which is consistent with literature reports of a short-term complication rate between 2.5% and 8% after TSA.^7,8,9,10,11^ We identified anemia as a risk factor for cardiac complications, renal complications, stroke/CVA, sepsis, UTI, postoperative anemia requiring transfusion, nonhome discharge, hospital readmission, and return to the surgical room within 30 days of TSA. Given the sheer volume of TSA procedures completed each year and an aging US population, it is reasonable to suggest that preoperative anemia status could contribute to notable morbidity after TSA in the years to come.^6^

Anemia is an identifiable and modifiable preoperative comorbidity that has been associated with functional decline, disability, physical strength, mortality, mental function, and various other medical comorbidities.^6,13,30,31^ In our study of 10,547 patients, 19.6% of the patients were found to be anemic, with 18.2% of the study population having mild anemia. In comparison to the normal hematocrit cohort, the mild anemia cohort had a greater proportion of patients who were women, older than the age of 70 years, and were afflicted by comorbidities including diabetes, chronic obstructive pulmonary disease, and hypertension. In addition, a greater proportion of patients with mild anemia were functionally dependent, used steroids, had an ASA ≥3, and had a BMI <25. These findings support previously identified relationships between anemia, patient functional status, and various other medical comorbidities. In our study, these notable associations between preoperative anemia status, patient demographics including age and sex, medical morbidities, and procedural characteristics were accounted for in multivariate regression modeling to evaluate the impact of preoperative anemia status in predicting short-term complications after TSA.

Anemia may result from nutrient deficiency, chronic proinflammatory states, chronic kidney disease, and bone marrow dysfunction.^12,13,31^ Deficiencies of vitamin B12, iron, and folate may account for one third of the etiologies of anemia.^12^ Given the findings of our study, preoperatively addressing these deficiencies and optimizing the patients' preoperative hematocrit level may reduce patient morbidity after TSA. Bisbe et al^32^ has developed a patient-specific algorithm for approaching the management of preoperative anemia before major orthopaedic surgery. However, the present study did not evaluate the impact of correcting patients' hematocrit preoperatively on postoperative complications after TSA. Thus, further research investigating the impact of optimizing patients' preoperative hematocrit levels on reducing complication rates after TSA is needed to further understand this relationship.

Shoulder arthroplasty continues to grow as a procedure in the field of upper extremity surgery. One factor contributing to its rapid annual increase in volume is the literature's support for reverse TSA as an effective treatment for elderly patients with Neer type 3 and 4 proximal humerus fractures.^33,34^ Given the prevalence of proximal humerus fractures in patients older than the age of 65 years, an increasing number of TSA procedures are projected to be done on many older individuals.^33,34^ In parallel, an increasing number of patients will be afflicted with complications after TSA in the years to come. Identifying and understanding relationships between modifiable preoperative risk factors, such as anemia, and postoperative complications may reduce the number of patients affected by complications after TSA. Furthermore, identifying various levels of preoperative anemia as risk factors for complication after TSA may also assist orthopaedic surgeons in risk stratification and preoperative patient counseling.

Our study is unique in that it stratifies anemia by level of severity and compares TSA outcomes among these groups. However, a few limitations exist that warrant discussion. The ACS NSQIP database only includes postoperative complications that occur within 30 days of the procedure. As intended, the results of this study are limited to the immediate 30-day postoperative period. In addition, this study was unable to distinguish between anatomic and reverse TSA, given the shared Current Procedural Terminology code between these procedures. It has been suggested that reverse TSA may have higher perioperative transfusion rates for anemia compared with anatomic TSA.^35^ Moreover, the etiology of each individual patients' anemia was also not available in the ACS NSQIP for analysis. Further research investigating the relationship between specific etiologies of anemia and their impact on postoperative complications after TSA would further elucidate this topic of interest. Previous studies have suggested that normal hematocrit levels may vary among different ethnicities.^36,37^ Beutler et al^36^ has proposed that the lower limits of normal hemoglobin concentrations differ between White and Black patients. This may represent an additional limitation of our study because our definition of anemia did not vary according to patients' ethnicities. Finally, data more specific to the field of orthopaedics, such as pain scores and functional outcomes, are not available through the ACS NSQIP database.

## Conclusions

A large number of patients undergoing TSA are anemic. This study used a large national database to identify preoperative anemia status as a risk factor for complications after TSA. In addition, this study identified a notable association between complication rates and increasing severity of preoperative anemia. Identification of anemia as a risk factor may assist orthopaedic surgeons in risk stratification and preoperative patient counseling. Additional research on the optimization of preoperative hematocrit levels and its impact on postoperative complications is needed to further understand this relationship.
